# Endoscopic versus surgical management of iatrogenic colonic perforations: a systematic review and meta-analysis

**DOI:** 10.1097/MS9.0000000000005388

**Published:** 2026-09-24

**Authors:** Yousef Mesaed Al-Shammari, Abdulateef Mohammad Atallah, Abdulrahman Salah Aldeen Alenezi, Abdulaziz Mohammad Abdullah Almuwaizri, Abdulaziz Mohammad Abdullah Alqouba, Abdulhadi Humoud Alasfour, Hashem Majed Alqallaf, Mohammed Ahmad Al-Awadhi, Saud Abdulaziz Aloun, Mohammed Al-Mahdi Al-Kurdi, Sulaiman Almazeedi

**Affiliations:** a Department of Ophthalmology, Al-Bahar Eye Center, Ibn Sina Hospital, Ministry of Health, Kuwait City, Kuwait; b Department of General Surgery, Jahra Hospital, Ministry of Health, Kuwait City, Kuwait; c Surgical Department, New Jahra Hospital, Ministry of Health, Kuwait City, Kuwait; d College of Medicine, University of Alexandria, Alexandria, Egypt; e Faculty of Medicine, University of Aleppo, Aleppo, Syria; f Department of Surgery, Jaber Al-Ahmad Hospital, Ministry of Health, Kuwait City, Kuwait

**Keywords:** colonic, iatrogenic, perforation, review, surgery

## Abstract

**Introduction:**

Iatrogenic colonic perforation is an uncommon but serious complication of colonoscopy that can lead to significant morbidity and mortality. While surgical management has traditionally been the standard of care, advances in endoscopic techniques have introduced less-invasive alternatives. However, the comparative effectiveness and safety of these approaches remain uncertain.

**Methods:**

A systematic review and meta-analysis were conducted in accordance with the PRISMA guidelines. PubMed, Scopus, and the Cochrane Library were searched from inception through January 2026. Studies comparing endoscopic and surgical management of iatrogenic colonic perforations were included. Data extraction and study selection were performed independently by two reviewers. Outcomes assessed included success rate, mortality, and length of hospital stay (LOS). Statistical analysis was conducted using a random-effects model, with results expressed as relative risks (RRs) or mean differences (MDs) with 95% confidence intervals (CIs).

**Results:**

Five retrospective cohort studies involving 160 patients were included. There was no statistically significant difference between endoscopic and surgical management in terms of success rate (RR = 0.81, 95% CI 0.65–1.01; *P* = 0.07) or mortality (RR = 0.39, 95% CI 0.10–1.65; *P* = 0.21). However, endoscopic management was associated with a significantly shorter LOS (MD = − 6.34 days, 95% CI −12.19 to −0.49; *P* = 0.02). Moderate heterogeneity was observed for success rate, while mortality showed no heterogeneity and LOS demonstrated substantial variability.

**Conclusion:**

Endoscopic management appears to be a safe and effective alternative to surgery in selected patients, offering the advantage of a reduced hospital stay. Nevertheless, surgical intervention remains necessary in more complex or delayed cases, highlighting the importance of appropriate patient selection.

## Introduction

Colonoscopy is a vital procedure that is both safe and effective for the diagnosis and treatment of colorectal diseases. Despite the safety and efficacy of the procedure, the increased number and complexity of colonoscopy cases have led to the recognized but rare complication of iatrogenic colonic perforation. Iatrogenic colonic perforation is defined as an unintentional colonic wall defect that results from a medical or surgical procedure^[^1^]^. The incidence rate of colonic perforation during colonoscopy varies from 0.1% to 0.8% for diagnostic and 0.15 to 3% for therapeutic colonoscopy^[^2–4^]^. Despite being a rare complication, colonic perforation is a clinically relevant adverse event that is associated with high rates of morbidity and mortality. This complication can lead to emergency surgery that includes bowel resection and the formation of a stoma. This complication can also lead to intra-abdominal sepsis, prolonged hospitalization, and death^[^3,5,6^]^.

HIGHLIGHTS

- Endoscopic management of iatrogenic colonic perforations demonstrates success rates comparable to those of surgery, with reduced morbidity and shorter hospital stays.
- Surgical intervention remains essential in cases of large perforations, delayed diagnosis, or failed endoscopic closure, thereby ensuring definitive management in complex cases.
- Meta-analytic findings suggest that endoscopic techniques are associated with lower complication rates and faster recovery than traditional surgical approaches.
- Early detection and appropriate patient selection are critical factors influencing outcomes and guiding optimal management strategies for colonic perforations.

Traditionally, iatrogenic colonic perforations have been treated surgically. The most common surgical intervention is laparotomy. However, recent improvements in endoscopic procedures have led to an increase in the minimally invasive treatment of colonic perforations. Endoscopic clipping is one such minimally invasive treatment for iatrogenic colonic perforation^[^7–9^]^. Endoscopic clipping was originally designed for the treatment of gastrointestinal bleeding. The device was successfully used to treat gastric perforations as well as colonic perforations^[^10^]^. The device works by approximating the edges of the perforation site to ensure immediate closure. This minimally invasive treatment may eliminate the need for surgical intervention^[^11^]^. Recent studies have confirmed the efficacy of endoscopic treatment for iatrogenic colonic perforation during both diagnostic and therapeutic procedures^[^12^]^.

Despite the increasing popularity of endoscopic treatment for colonic perforation, the efficacy of endoscopic management for iatrogenic colonic perforation remains unclear. Reported outcomes have been variable. Differences in results may be attributed to patient selection, perforation site, time to diagnosis, device choice, and operator expertise. Accordingly, a comprehensive overview of the existing literature is necessary to clarify efficacy. Therefore, this meta-analysis aims to evaluate the efficacy and safety of endoscopic management for iatrogenic colonic perforation.

## Methods

This systematic review and meta-analysis was conducted in accordance with the PRISMA guidelines, the AMSTAR-2 checklist, and the TITAN checklist ^[^13–15^]^. This systematic review and meta-analysis was registered in PROSPERO under registration number CRD420261327627.

### Search strategy and eligibility criteria

A literature search was performed to identify studies comparing endoscopic and surgical management of iatrogenic colonic perforations. The electronic databases PubMed, Scopus, and the Cochrane Library were searched from their inception through January 2026. The search strategy combined Medical Subject Headings (MeSH) terms and free-text keywords related to “iatrogenic colonic perforation,” “colonoscopic perforation,” “endoscopic management,” “endoscopic closure,” “surgical management,” “laparotomy” and “laparoscopy.” No language restrictions were applied. Additionally, the reference lists of included articles and relevant reviews were manually screened to identify any additional eligible studies.

Studies were considered eligible if they met the following criteria: (1) included patients with iatrogenic colonic perforations; (2) compared endoscopic management with surgical management; and (3) reported at least one of the predefined outcomes of interest. Prospective cohort studies and retrospective comparative studies were included. Case reports, case series without a comparator group, reviews, editorials, conference abstracts without full text, and studies not reporting relevant outcomes were excluded.

### Study Selection and Data Extraction

Two reviewers independently screened the titles and abstracts of all retrieved records. Full-text articles were subsequently reviewed to confirm eligibility according to the predefined inclusion and exclusion criteria. Any discrepancies between reviewers were resolved through discussion and, when necessary, consultation with a third reviewer. Data were extracted independently by two reviewers using a standardized data collection form. The extracted data included study ID, country, study design, sample size, patient demographics, and location of perforation. The primary outcomes of interest were the success rate, defined as successful closure or resolution of the perforation without the need for additional intervention; the mortality rate; and the length of hospital stay (LOS). These outcomes were selected to comprehensively evaluate both the effectiveness and safety of endoscopic versus surgical management.

### Quality assessment

The methodological quality of included non-randomized studies was assessed using the Newcastle–Ottawa Scale (NOS). Two reviewers independently evaluated each study across the three domains of the NOS: selection of study groups, comparability of groups, and assessment of outcomes. Studies were assigned a score based on the number of stars achieved in each domain. Disagreements were resolved by consensus or consultation with a third reviewer. Study quality was considered in the interpretation of the pooled results.

### Statistical analysis

Statistical analyses were performed using R software (version 4.1.2) with the *metafor* package (version 4.8-0). Dichotomous outcomes (success rate and mortality rate) were pooled as relative risks (RRs) with 95% confidence intervals (CIs) using the generic inverse-variance method, while the continuous outcome (length of hospital stay) was summarized as mean differences (MDs) with 95% CIs. A random-effects model was applied to account for anticipated clinical and methodological heterogeneity among studies. Statistical heterogeneity was assessed using the tau-squared (τ^2^) statistic and quantified using the *I*^2^ statistic. An *I*^2^ value greater than 50% was considered indicative of moderate heterogeneity, while values exceeding 75% were considered indicative of substantial heterogeneity. Statistical significance was defined as a two-sided *P*-value < 0.05. Sensitivity analyses were conducted by sequentially excluding individual studies to evaluate the robustness of the pooled estimates.

## Results

### Literature Search Results

The literature search resulted in 182 records from three major databases: PubMed (*n* = 94), Scopus (*n* = 61), and Cochrane (*n* = 27). After removing duplicates, 148 unique records remained. Titles and abstracts were screened according to PRISMA guidelines, and 30 records were selected for full-text review. Following a detailed full-text assessment, five studies met the inclusion criteria and were included in our review. The study selection process is shown in Figure 1.

**Figure 1.**
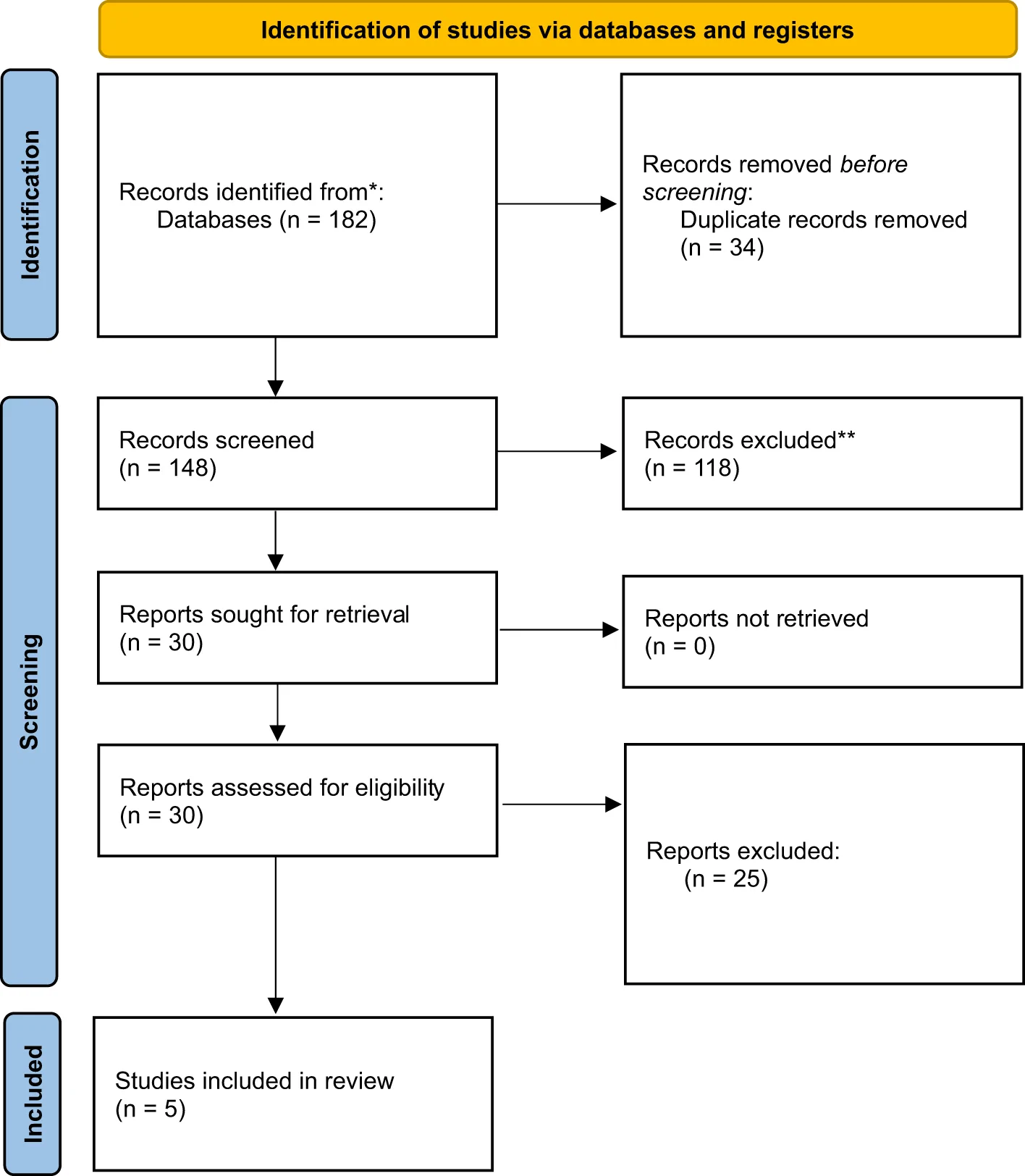
PRISMA flow chart of the selection process.

### Characteristics of the included studies

Our review included five retrospective cohort studies published between 2012 and 2026, comprising a total of 160 patients with iatrogenic colonic perforation^[^16–20^]^. All included studies were single-country retrospective cohort studies conducted in Greece, Turkey, Korea (two studies), and Malaysia. The number of patients included ranged from 12 to 56 per study. In addition, the mean age of participants was generally in the 60s, ranging from 62.3 to 69.3 years across study groups. The sex distribution revealed that all studies reported a predominance of female patients, with the percentage of female participants ranging from 32.4% to 90.9%. The sigmoid colon and rectosigmoid junction were the most common locations of perforation, accounting for 57.1% to 91.7% of cases. Finally, the reported hospital iatrogenic perforation rate ranged from 0.0002 to 0.0017. Table 1 summarizes the detailed characteristics of the included studies.

**Table 1:** Characteristics of the included studies.

Study ID	Country	Study design	Mean age	Number of patients	% Female	Location of Perforation	Hospital iatrogenic perforation rate	Aim	Conclusion
Paspatis *et al* [17]	Greece	Retrospective cohort study	Before 2012: 67.6 (12.7)	Before 2012: 15	Before 2012: 86.7%	Sigmoid colon (60%)	0.0004	Compare the management of iatrogenic perforations before vs after introduction of clipping.	Advanced clipping significantly reduced surgery rate and hospital stay.
			After 2012: 69.1 (9.5)	After 2012: 11	After 2012: 90.9%				
Çetinkaya *et al* [18]	Turkey	Retrospective cohort study	62.3 (12.1)	Endoscopic: 8	0.667	Sigmoid colon (57.1%)	0.00047	Evaluate clinical features, management, and outcomes of ICP with emphasis on clip closure.	Clip closure effective in selected small, early-recognized perforations; surgery remains essential when closure fails or diagnosis is delayed.
				Surgery: 34					
Cha *et al* [16]	Korea	Retrospective cohort study	Endoscopic: 69.3 (11.2)	Endoscopic: 22	Endoscopic: 68.2%	Rectosigmoid (84.8%)	0.0009	Compare characteristics and outcomes of diagnostic vs therapeutic ICP and assess indication for endoscopic repair.	Immediate detection and small perforation size are key predictors of successful endoscopic repair.
			Surgery: 65.9 (11.5)	Surgery: 34	Surgery: 32.4%				
Chan *et al* [20]	Malaysia	Retrospective cohort study	Endoscopic: 68 (21.1)	Endoscopic: 7	Endoscopic: 71.4%	Rectosigmoid (91.7%)	0.0017	Compare clinical outcomes and costs of endoscopic clipping vs surgery in ICP.	Endoscopic clipping may be more cost-effective than surgery.
			Surgery: 63.8 (17.6)	Surgery: 5	Surgery: 40%				
Kim *et* *al*^[^19^]^	Korea	Retrospective cohort study	Endoscopic: 66.9 (12.3)	Endoscopic: 13	Endoscopic: 61.5%	Sigmoid colon (59.3%)	0.0002	Evaluate frequency and effectiveness of endoscopic clip closure vs surgery for diagnostic ICP.	Endoscopic clip closure is effective for diagnostic colonoscopy-related perforations.
				Surgery: 11	Surgery: 54.5%				
			Surgery: 68.1 (10.8)						

### Quality assessment

The quality of the included studies was assessed using the NOS. Paspatis *et al*[17], Çetinkaya *et al*[18], Cha *et al*[16], and Kim *et al* showed high methodological quality, with scores of 8–9 stars. Chan *et al*[20] showed moderate-to-high quality, with 7 stars. All studies included clearly defined patient groups and reliable hospital records. Outcomes were clearly reported, and follow-up was adequate in all studies, with little or no loss to follow-up. The main limitation was the retrospective design of all studies. Most studies did not adjust for possible confounding factors. Overall, the risk of bias was low, and the included studies provided reliable evidence (Table 2).

**Table 2:** Quality assessment.

Study ID	Selection	Comparability	Outcome	Total
Paspatis *et al* [17]	4	1	3	8
Çetinkaya *et al* [18]	4	1	3	8
Cha *et al* [16]	4	2	3	9
Chan *et al* [20]	3	1	3	7
Kim *et al*	3	2	3	8

### Meta-analysis

#### Success rate

The analysis of five studies including 160 patients with iatrogenic colonic perforation revealed that endoscopy did not show a statistically significant difference in success rate compared with surgery (Fig. 2: RR = 0.81, 95% CI [0.65, 1.01], *P* = 0.07). Moderate-to-high heterogeneity was detected across the included studies (tau^2^: = 0.49, *I*^2^: = 48.47%). Sensitivity analysis using the leave-one-out method revealed that the exclusion of Cha *et al* [16] significantly reduced heterogeneity across studies (*I*^2^: = 1.9%).

**Figure 2.**
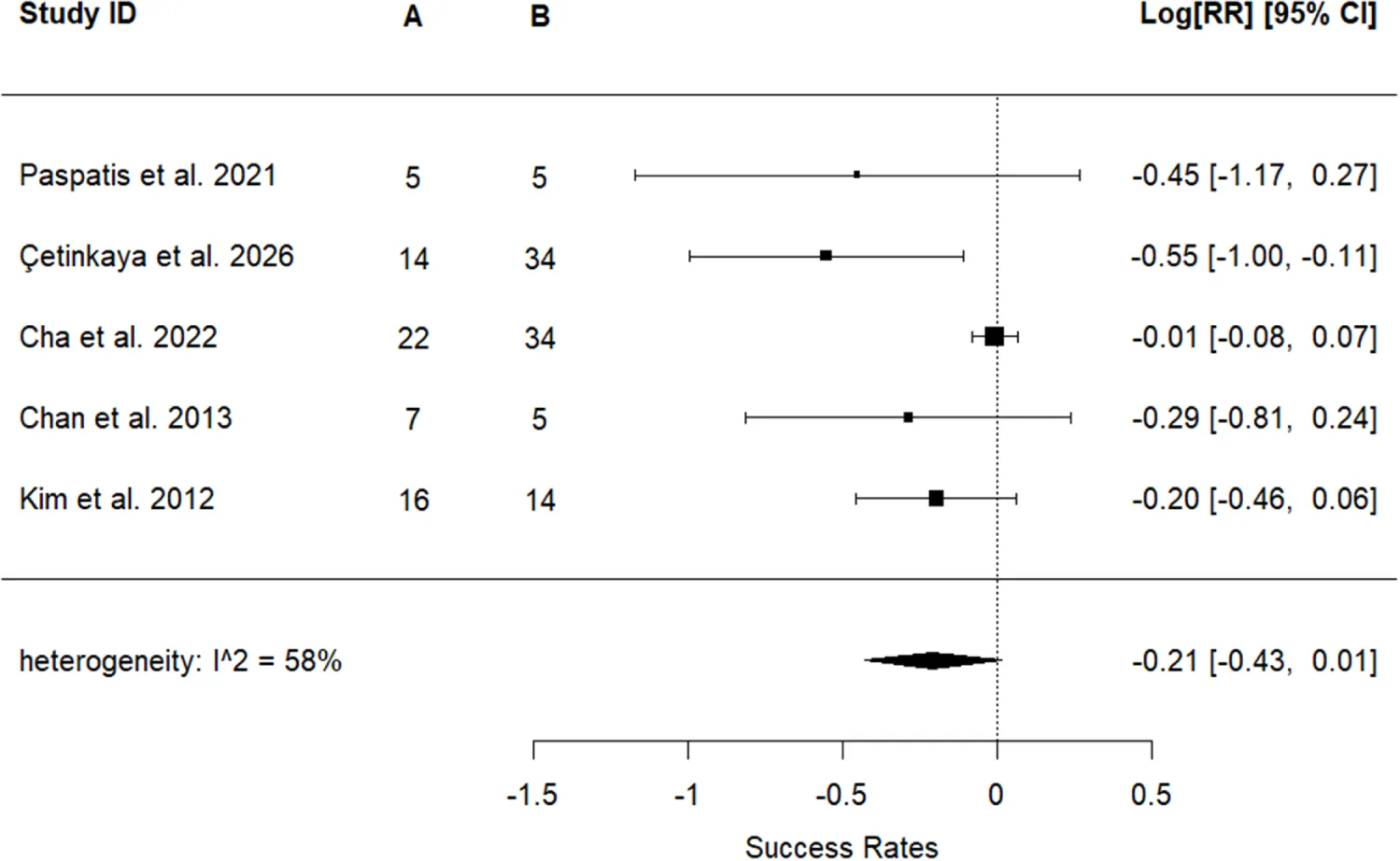
Meta-analysis of the success rate.

#### Mortality rate

The analysis of five studies including 160 patients with iatrogenic colonic perforation revealed that endoscopy did not show a statistically significant difference in mortality rate compared with surgery (Fig. 3: RR = 0.39, 95% CI [0.10, 1.65], *P* = 0.21). Very low heterogeneity was detected across the included studies (tau^2^: = 0.0, *I*^2^: = 0.0%). The sensitivity analysis using the leave-one-out method revealed no statistically significant difference when any of the included studies was excluded.

**Figure 3.**
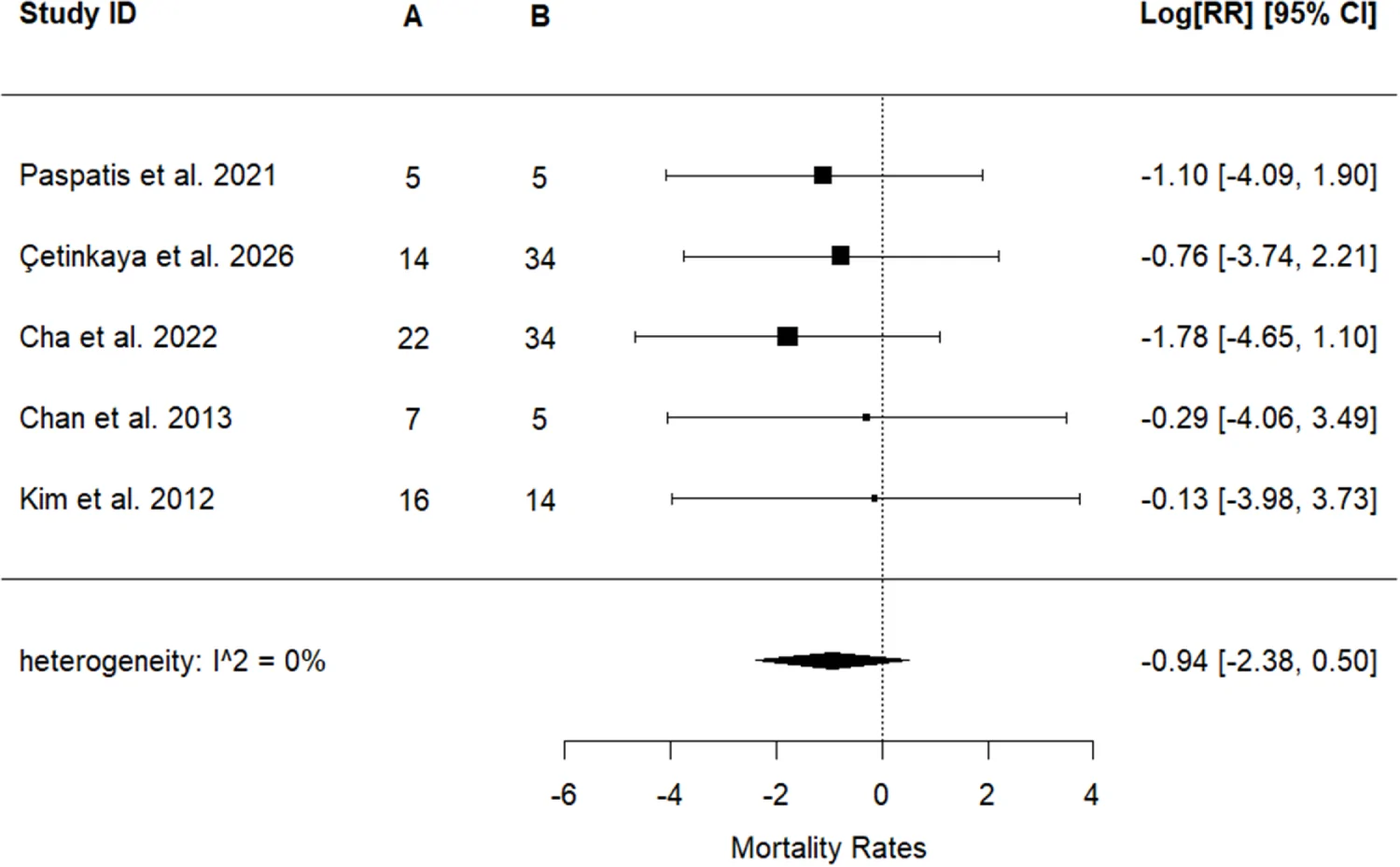
Meta-analysis of the mortality rate.

#### Length of stay

The analysis of three studies that included 92 patients with iatrogenic colonic perforation revealed that endoscopy was associated with a statistically significant shorter length of stay compared with surgery (Fig. 4: MD = − 6.34 days; 95% CI, −12.19 to − 0.49; *P* = 0.02). High heterogeneity was detected across the included studies (tau^2^: = 17.5; *I*^2^: = 66.38%).

**Figure 4.**
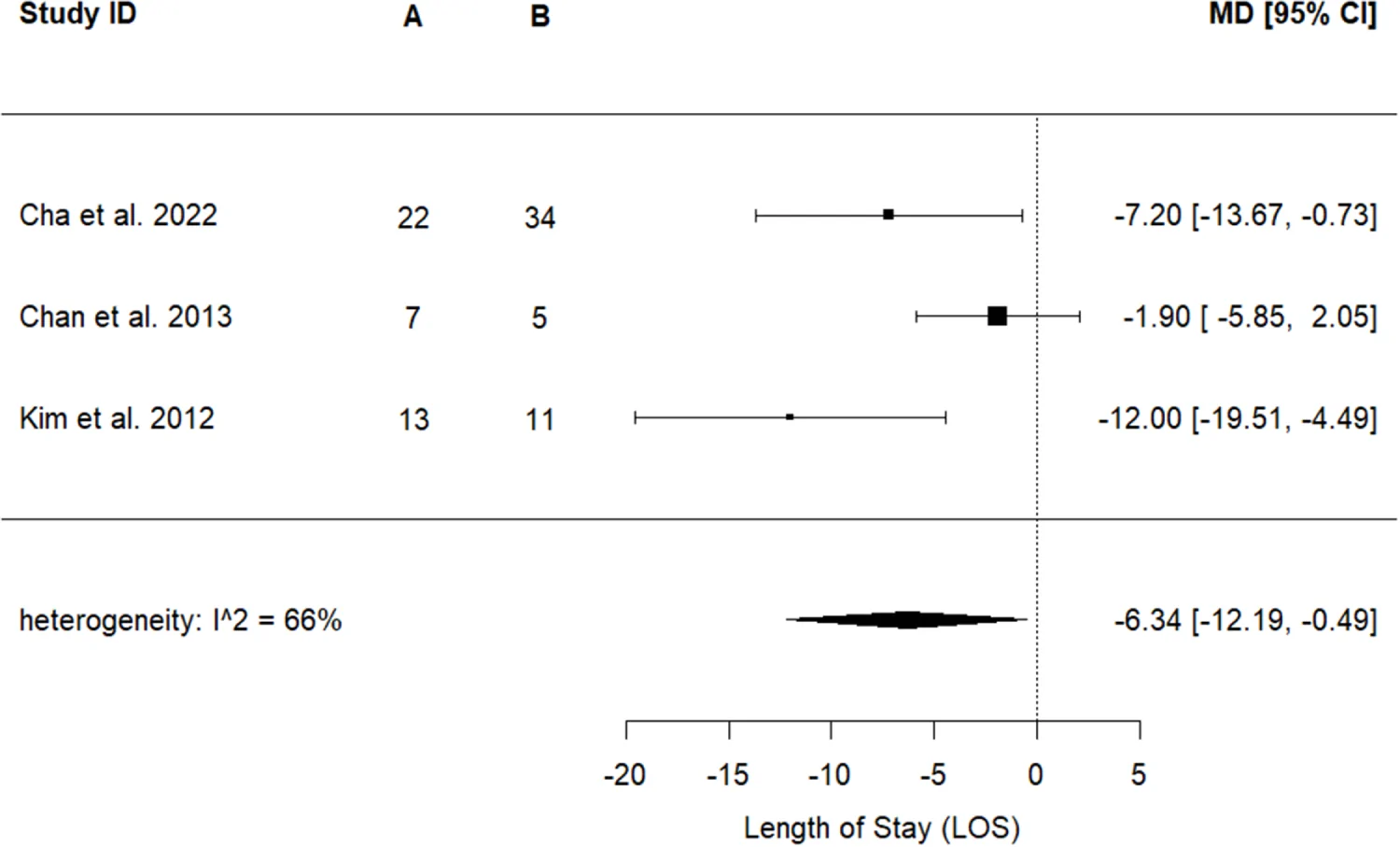
Meta-analysis of length of stay (LOS).

### Qualitative analysis

#### Treatment failure and surgical intervention

Several studies reported treatment failure and the need for surgery. The failure rate of endoscopic clipping ranged from 19% to 43% among patients in whom clipping was attempted^[^18,19^]^. The overall proportion of patients requiring surgical management ranged from 51% to 81%, depending on the study and patient selection^[^16,18^]^. Surgical procedures varied in severity. They included primary repair, bowel resection, and stoma formation.

#### Recovery outcomes and resource utilization

Other recovery-related outcomes were also described. Time to oral intake was shorter in the endoscopic group when reported^[^18^]^. Healthcare costs were lower with endoscopic treatment compared with surgery^[^16^]^. Overall, successful endoscopic treatment was associated with faster recovery and lower resource use, whereas surgery was required for larger or delayed perforations.

## Discussion

This systematic review and meta-analysis provides the most recent evidence comparing endoscopic and surgical management of iatrogenic colonic perforations. Iatrogenic colonic perforation is rare but serious; it occurs in 0.02–0.8% of diagnostic colonoscopies and can reach 3% or more in therapeutic procedures^[^16,19^]^. Although rare, it can cause serious complications and may require surgery.

It can prolong hospital stays. It also carries a risk of death^[^16^]^. Surgery has been the standard treatment. But new endoscopic clipping techniques offer a less invasive option. They show promising success rates^[^18,19^]^. However, the optimal management strategy remains controversial, and clear evidence comparing these approaches is still needed.

Our analysis revealed that no significant difference in success rates was found between endoscopy and surgery (RR = 0.81, *P* = 0.07; *I*^2^ = 48.47%). This finding was consistent across the five individual studies included in our analysis, except for the study by Çetinkaya *et al*[18]. Çetinkaya *et al*[18] showed that surgical treatment had a better overall success rate. Çetinkaya *et al*[18] reported better outcomes with surgery, mainly because a large proportion of their patients had delayed diagnosis or larger perforations, in which clip closure was either not attempted or failed. In addition, the study was conducted in a resource-limited setting where advanced closure devices were not routinely available, limiting endoscopic success^[^18^]^. The heterogeneity arose because the included studies reported markedly different endoscopic success rates and proportions of patients undergoing primary surgery, resulting in variation in the calculated effect sizes across cohorts^[^18–20^]^. Across the five included studies, endoscopic clip closure demonstrated high success rates when perforations were recognized early and were small^[^16–20^]^. In contrast, surgery remained the most frequently applied treatment, particularly in cases of delayed diagnosis or larger defects^[^16,17^]^. Accordingly, careful patient selection and early recognition appear to be critical factors in achieving comparable outcomes between endoscopic and surgical management.

Our analysis revealed no significant difference in the mortality rate between endoscopy and surgery (RR = 0.39, *P* = 0.21, *I*^2^ = 0.0%), and this finding was consistent across all five individual studies included in our analysis, as none demonstrated a mortality difference between the two treatment groups. In several cohorts, no deaths occurred among patients managed endoscopically^[^19^]^, whereas mortality events were reported among surgically treated patients^[^17^]^. The absence of statistical heterogeneity (*I*^2^ = 0.0%) reflects the low mortality rates reported across studies, regardless of the management strategy. Overall, these findings suggest that endoscopic management does not increase mortality risk compared with surgery.

Our analysis revealed that endoscopy was associated with a significantly shorter length of stay compared with surgery (MD = − 6.34 days, *P* = 0.02, I^2^ = 66.38%). Several studies also reported shorter hospital stays in patients successfully treated with endoscopic clipping ^[^17,18^]^. However, surgically treated patients often require longer postoperative recovery and monitoring^[^16^]^. The heterogeneity likely reflects variability in surgical approaches (open vs. laparoscopic) and the inclusion of patients requiring reoperation after failed endoscopic attempts^[^17,18^]^. Overall, these results suggest that, when appropriate, endoscopic treatment allows faster recovery and earlier discharge than surgery.

The exclusion of the study by Cha *et al*[16] reduced heterogeneity, suggesting that the study had an impact on the high level of heterogeneity. The main differences between the study and the others were that it included both locally treated and referred patients and that it analyzed a heterogeneous cohort of diagnostic and therapeutic colonoscopy-related perforations. These differences in methodology and clinical data could have led to effect estimates that were inconsistent with those of the other studies; hence, the high level of heterogeneity.

Chan *et al*[20] reported that endoscopic clipping was associated with lower costs compared with surgery (US$115.10 vs. US$ 1479.50; *P* = 0.012), as well as lower investigation costs (US$124.60 vs. US$512.90; *P* = 0.048). There was also a trend toward reduced overall inpatient costs in patients managed endoscopically (US$ 1481.70 vs. US$ 3281.90). These differences are explained by the avoidance of operating room expenses, anesthesia, and the more extensive postoperative care typically required after surgical intervention. The available evidence suggests that, when technically successful, endoscopic closure offers a more cost-effective strategy without compromising clinical outcomes^[^20^]^.

This meta-analysis shows that endoscopic treatment is safe and effective in selected patients. It can be a good alternative to surgery, although surgery is still necessary in some cases. Endoscopic clipping has success and mortality rates similar to those of surgery; it also shortens hospital stay and may reduce costs. Treatment should be individualized, and early diagnosis and patient stability should guide decision-making.

A previous meta-analysis comparing laparoscopic and open surgery demonstrated that laparoscopic surgery resulted in significantly fewer postoperative complications compared with open surgery (18.2% vs. 53.5%). Laparoscopy was also associated with a shorter hospital stay (MD = − 5.35 days). There was no significant difference in mortality or reintervention rates between the two approaches. The conversion rate from laparoscopy to open surgery was 10%. These results suggest that minimally invasive surgical management improves recovery without compromising safety^[^21^]^.

Current guidelines for the management of ICP suggest that management should be tailored to the timing of the diagnosis, the nature of the perforation, and the patient's condition. Conservative management is recommended for patients who are hemodynamically stable without symptoms of generalized peritonitis. This includes bed rest, antibiotics, and observation. If the perforation is identified early (within 4 hours), endoscopic closure using clips is recommended, especially for small perforations (<1 cm). Surgery is recommended in patients with peritonitis, deterioration in condition, a large perforation, or failure of conservative management. Laparoscopic surgery is preferred over open surgery due to lower morbidity^[^12^]^. Figure 5

**Figure 5.**
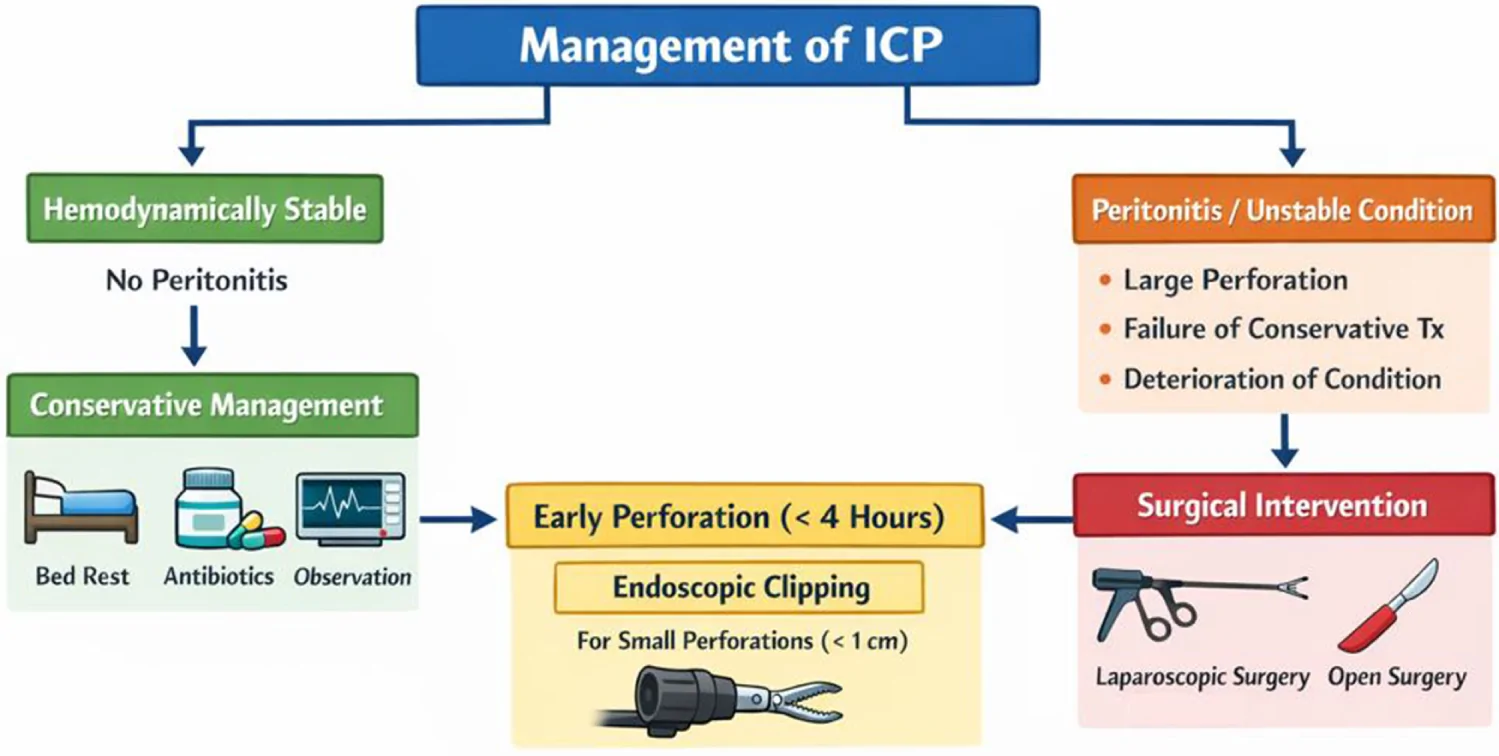
Management of ICP.

From a clinical practice perspective, the treatment of iatrogenic colonic perforation should mainly depend on the time of diagnosis, the size of the perforation, and the patient’s condition. In fact, endoscopic closure of the perforation should be attempted in cases where the perforation is detected during colonoscopy or shortly after the procedure in hemodynamically stable patients without signs of generalized peritonitis. On the other hand, a surgical approach should be preferred in cases of delayed diagnosis, large perforation size, signs of generalized peritonitis, or failure of endoscopic closure. In such cases, it is very important to perform surgical intervention at an appropriate time to avoid further deterioration of the patient’s condition.

This study has several limitations. All included studies were retrospective in design, which increases the risk of selection bias and confounding. The sample sizes were relatively small, and the reporting of perforation characteristics was not uniform across studies. In addition, the surgical groups employed different operative techniques, such as laparoscopic and open surgery. Another important limitation is associated with selection bias. It is common for patients undergoing endoscopic clipping to have more favorable conditions, such as smaller perforations and earlier detection. However, patients with more severe conditions, such as sepsis, delays in diagnosis, and advanced age, have a higher likelihood of undergoing surgical management. This might have contributed to the increased length of stay in the surgical group, not because of the treatment itself but because of patient characteristics.

Future prospective multicenter studies with standardized definitions of perforation size, timing of diagnosis, and treatment algorithms are needed. The development of large international registries would help refine patient selection criteria and establish clearer evidence-based guidelines for the optimal management of iatrogenic colonic perforations.

## Conclusion

This meta-analysis showed that endoscopic treatment is a safe and effective option for iatrogenic colonic perforation. There was no significant difference in success rates compared with surgery. There was also no significant difference in mortality. Endoscopic treatment was associated with a shorter hospital stay. Patients treated endoscopically recovered faster. This suggests a clinical advantage in selected cases. However, endoscopic closure is not suitable for all patients. It is more effective when perforations are small. It works best when the diagnosis is made early. Patient stability is very important. Surgery remains necessary in complicated cases. It is required for large perforations or a delayed diagnosis. It is also needed when endoscopic treatment fails. Overall, endoscopic management can be considered a first option in carefully selected patients, while surgery continues to play a crucial role in more severe cases.

## Data Availability

All data are available within the manuscript and its supplemental files.

## Research registration unique identifying number

PROSPERO registration (CRD420261327627)
